# Supplementary material for: Physics-aware differentiable design of magnetically actuated kirigami for shape morphing
Source: Nat Commun. 2023 Dec 21;14:8516. doi: 10.1038/s41467-023-44303-x (PMC10739944; doi:10.1038/s41467-023-44303-x)
Supplement: Supplementary file 1 — Supplementary Information [file 41467_2023_44303_MOESM1_ESM.pdf]

Supplementary Information for
Physics-aware differentiable design of magnetically actuated kirigami for shape morphing

Liwei Wang, Yilong Chang, Shuai Wu, Ruike Renee Zhao, Wei Chen

Corresponding author

Correspondence to: Wei Chen (weichen@northwestern.edu)

This PDF file includes:

Supplementary Note S1 to S7

Supplementary Figure S1 to S9

Supplementary References

Other supporting materials for this manuscript include the following:

Supplementary Movie S1 to S8

Source Data file

Contents of Supplementary Note

Supplementary Note 1. Geometrical constraints 2

S1.1 Edge compatibility 3

S1.2 Angle compatibility 3

S1.3 Non-overlap constraint 4

S1.4 Deployed shape constraint 5

S1.5 Rectangular compact shape constraint 5

S1.6 Aspect ratio constraint of compact shape 5

S1.7 Rigid deployable constraint 6

S1.8 Two-way rigid contractible constraint 6
Supplementary Note 2. Differentiable kinematic analysis 7
Supplementary Note 3. Finite element analysis 11
Supplementary Note 4. Hyper-elastic hinge model 11
Supplementary Note 5. Differentiable magneto-elastic energy model and energy-based simulation 13
S5.1 Elastic energy model 14
S5.2 Modified elastic energy model 15
S5.3 Magnetic energy model 16
S5.4 Energy-based simulation 17
Supplementary Note 6. Physics-aware kirigami optimization 21
S6.1 Optimization with one compact state 21
S6.2 Two-way contractible design 24
Supplementary Note 7. Printing of the magnetic kirigami 29
Supplementary References 30

Supplementary Note 1. Geometrical constraints

We incorporate kinematic feasibility as differentiable geometric constraints in our kirigami optimization framework, following the work of Choi et al[1]. Herein, we provide a concise overview of each geometric constraint to ensure the comprehensiveness of our study. Note that, since the optimization is performed on the deployed configuration, we need to impose all the constraints only on the deployed configuration to facilitate the optimization process. For ease of illustration, we will use V_i to present node i with coordinate $\mathbf{x}_i = [x_i, y_i]^T$, E_{ij} to represent the edge between V_i and V_j with length $l_{ij} = \|\boldsymbol{x}_i - \boldsymbol{x}_j\|$, and P_{ijkl} to represent the panel whose boundary is formed by sequentially connecting $(E_{ij}, E_{jk}, E_{kl}, E_{li})$. If node V_i is a hinge, we use $\alpha_{i,1}$ and $\alpha_{i,2}$ to represent the two angles belonging to two different panels but connected by V_i in the deployed state. In contrast, if the node V_i is not a hinge (i.e., nodes on the boundary), α_i is used to represent the angle of the panel at the node V_i. These angles are within the range of $(0,2\pi)$. When there is

only one compact state, we will use S_0 to represent the deployed state and S_- to represent the compact state. When there are two compact states in the two-way contractible designs, we will use S_- , S_0, and S_+ to represent negative, zero, and positive states, respectively. We use negative/positive signs in the left superscripts for entities in states S_- and S_+, respectively. For example, ${}^{-}V_i$ represents node i in the negative state S_-.

S1.1 Edge compatibility

In the deployed state, the cutting line between panels in a compact kirigami expands into voids, resulting from the counter-rotation of the panels (Fig. S1a). To maintain edge compatibility, we identify a set of edge pairs $A = \{(E_{ij}, E_{ik})\}$ with a shared node V_i and overlapping in the compact state, i.e., ${}^{-}E_{ij} = {}^{-}E_{ik}$ (indicated by the same color in Fig. S1a). The edge compatibility constraint can be given as

$$(l_{ij} - l_{jk})^2 = 0, \text{ for all } (E_{ij}, E_{ik}) \in A. \tag{S1}$$

It enforces that each pair of edges in A has an equal length, ensuring edge compatibility during the design process.

S1.2 Angle compatibility

In the compact state, every four neighboring panels will share one node in the cutting, as shown in Fig. S1b. Assume that the shared node ${}^{-}V_i({}^{-}V_j)$ in the compact state correspond to nodes V_i and V_j in the deployed state, we can identify all these node pairs $C = \{(V_i, V_j)\}$. To maintain angle compatibility, the sum of angles around the shared node ${}^{-}V_i({}^{-}V_j)$ should be equal to 2π. Note that this angle sum requirement is also preserved in the deployed state, we can obtain another geometrical constraint on the deployed configuration,

$$\alpha_{i,1} + \alpha_{i,2} + \alpha_{j,1} + \alpha_{j,2} - 2\pi = 0, \qquad \text{for all } (V_i, V_j) \in C. \tag{S2}$$

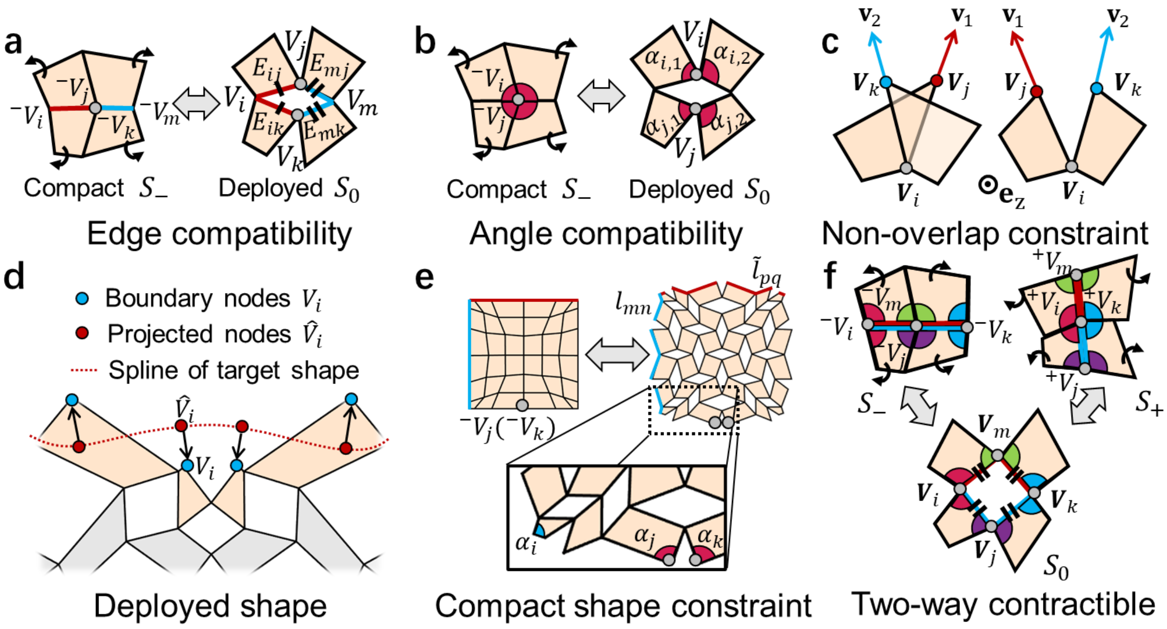

Supplementary Figure 1. Geometric constraints. **a** Edge compatibility constraint, **b** Angle compatibility constraint, **c** Non-overlap constraint, **d** Target deployed shape constraint, **e** Compact shape constraint, **f** Two-way contractible constraint. All these constraints are imposed on the deployed/zero state (S_0).

S1.3 Non-overlap constraint

Panels in the deployed state should not overlap with each other. To ensure this, as shown in Fig. S1c, at every angle formed by edges E_{ij} and E_{ik} between two adjacent panels, the following non-overlap constraints should be satisfied:

$$\mathbf{v}_1 \times \mathbf{v}_2 \cdot \mathbf{e}_z = \left(\mathbf{x}_j - \mathbf{x}_i\right) \times \left(\mathbf{x}_k - \mathbf{x}_i\right) \cdot \mathbf{e}_z < 0. \tag{S3}$$

Herein, we substitute the two edges vectors into $\mathbf{v}_1$ and $\mathbf{v}_2$ in a way that the ordered triplet $(\mathbf{v}_1, \mathbf{v}_2, -\mathbf{e}_z)$ forms a right-handed set, i.e., $\mathbf{v}_1 \times \mathbf{v}_2 \cdot (-\mathbf{e}_z) > \mathbf{0}$ in the deployed state of a regular cutting (Fig. 1b). $\mathbf{e}_z = (0,0,1)$ is the unit vector pointing out-of-the-plane.

S1.4 Deployed shape constraint

As shown in Fig. S1d, we use a spline to represent the target shape in the deployed state. To achieve the target shape, boundary nodes V_i of the kirigami should lie on the spline, which can be imposed as an equality constraint as

$$\|\mathbf{x}_i - \hat{\mathbf{x}}_i\|^2 = 0, \tag{S4}$$

where $\mathbf{x}_i$ is the coordinate of V_i and $\hat{\mathbf{x}}_i$ is the coordinate of the point $\hat{V}_i$ on the spline projected from V_i. $\hat{V}_i$ can be efficiently found by searching for the point on the spline with a minimal distance to V_i.

S1.5 Rectangular compact shape constraint

As an optional constraint, we can further restrict the shape in the compacted state to a rectangle. This can be achieved by adding two sets of constraints, as shown in Fig. S1e. The first set of constraints is imposed on the four corner angles of the kirigami to make them right angles

$$\alpha_i - \frac{\pi}{2} = 0. \tag{S5}$$

The second set of constraints is imposed on each pair of angles α_j and α_k on the boundary with a shared node $^-V_j(^-V_k)$ in the compact space

$$\alpha_j + \alpha_k - \pi = 0. \tag{S6}$$

S1.6 Aspect ratio constraint of compact shape

This is also an optional constraint, which restricts the ratio between the height and width of a rectangular compact kirigami to be a given value a, which can be formulated as

$$\sum l_{mn} - a \sum \tilde{l}_{pq} = 0, \tag{S7}$$

where l_{mn} and $\tilde{l}_{pq}$ are the lengths of each edge on the left and top boundaries of the compact kirigami, respectively (Fig. S1e).

S1.7 Rigid deployable constraint

As illustrated in the main text, this constraint enforces the angles around any rotating hinge (marked in the same color in Fig. 2d-f) in an enclosed cutting sum up to π. This can ensure there is no geometrical frustration during the deployment process. It should be noted that, kirigami satisfying this rigid deployable constraint will automatically satisfy the angle compatibility constraint in equation (S2). Therefore, we don't need to include the angle compatibility constraint (equation (S2)) for all the rigid-deployable kirigami in this study.

S1.8 Two-way rigid contractible constraint

This constraint is imposed for two-way rigid contractible designs in Fig. 6. As shown in Fig. S1f, the goal is to ensure the deployed kirigami has two valid compact states, so that the kirigami can follow two kinematically feasible paths to reach any of the two states. To achieve this, we first need to add extra angle constraints[2]

$$\alpha_{i,1} + \alpha_{i,2} - \pi = 0, \tag{S8}$$

where $\alpha_{i,1}$ and $\alpha_{i,2}$ are the two angles belonging to different panels but connected by the same hinge V_i in the deployed state (angles with the same color in Fig. S1f). When equation (S8) is imposed, we can ignore the rigid deployable constraint, which is simply a special case for equation (S8). Besides angle constraints, we also need to add extra edge constraints at each hinge V_i to ensure two-way rigid contraction. Specifically,

we identify a set of edge pairs $D = \{(E_{ij}, E_{im})\}$ with a shared node V_i and overlapped either in the negative ($^{-}E_{ij} = {}^{-}E_{im}$) or positive states ($^{+}E_{ij} = {}^{+}E_{im}$). We can then impose extra edge constraints as

$$(l_{ij} - l_{im})^2 = 0, \text{ for all } (E_{ij}, E_{im}) \in D. \tag{S9}$$

Similarly, when equation (S9) is imposed, we can ignore the previous edge compatibility constraint (equation (S1)), which is a special case for equation (S9), i.e., $A \subset D$.

Supplementary Note 2. Differentiable kinematic analysis

In our design framework, the key to incorporating physics into the design process is to ensure the differentiability of the kinematic analysis, so that we can obtain the gradient information of kirigami configurations at different states with respect to the design variables. As illustrated in the main text and Fig. 3, we adopt a sequential process to update the node positions while respecting the kinematic constraints with the preceding nodes. This sequential approach entails applying a series of analytical transformations in a predetermined order. By leveraging the chain rule, we can calculate the analytical gradient of nodal locations with respect to the deployed angle without difficulty.

Herein, we will perform several steps of this sequential kinematic analysis to provide a clearer illustration. We focus on solving the nodal location for the panels located in the middle two columns, as depicted in Fig. S2. Since the kirigami is symmetric, we only need to work on the right column and then update the left column by reflection. Assume that the kirigami cutting is given in a state with $X_0 = \{({}^{0}x_i, {}^{0}y_i)\}$ corresponding to a deployed angle θ_0, we aim to update the nodal locations to obtain configuration in a new state $X = \{({}^{t}x_i, {}^{t}y_i)\}$ corresponding to a deployed angle $\theta_{\mathrm{t}} = \theta_0 + \Delta\theta$. As shown in Fig. S2b-c, we begin from the topmost panel, using node $({}^{0}x_1, {}^{0}y_1)$ as the fixed reference point, i.e., $({}^{\mathrm{t}}x_1, {}^{\mathrm{t}}y_1) = ({}^{0}x_1, {}^{0}y_1)$. Based on the reference point and rotated angle $\Delta\theta$, We can easily obtain the updated positions for other nodes in this panel by multiplying a rotational matrix

$$\begin{bmatrix} {}^{\mathrm{t}}x_i \\ {}^{\mathrm{t}}y_i \end{bmatrix} = \begin{bmatrix} {}^{0}x_1 \\ {}^{0}y_1 \end{bmatrix} + \begin{bmatrix} \cos\left(\frac{\Delta\theta}{2}\right) & -\sin\left(\frac{\Delta\theta}{2}\right) \\ \sin\left(\frac{\Delta\theta}{2}\right) & \cos\left(\frac{\Delta\theta}{2}\right) \end{bmatrix} \cdot \begin{bmatrix} {}^{0}x_i - {}^{0}x_1 \\ {}^{0}y_i - {}^{0}y_1 \end{bmatrix}$$

$$= \begin{bmatrix} {}^{0}x_1 + \left({}^{0}x_i - {}^{0}x_1\right)\cos\left(\frac{\Delta\theta}{2}\right) - \left({}^{0}y_i - {}^{0}y_1\right)\sin\left(\frac{\Delta\theta}{2}\right) \\ {}^{0}y_1 + \left({}^{0}x_i - {}^{0}x_1\right)\sin\left(\frac{\Delta\theta}{2}\right) + \left({}^{0}y_i - {}^{0}y_1\right)\cos\left(\frac{\Delta\theta}{2}\right) \end{bmatrix}, \text{ for } i = 2,3,4. \tag{S10}$$

This equation can be considered a transformation mapping $X_1 = f_1(X_0, \Delta\theta)$ from the initial state X_0 and deployed angel change $\Delta\theta$ to a partially updated state $X_1 = \{({}^{\mathrm{t}}x_i, {}^{\mathrm{t}}y_i) | i = 1,2,3,4\}$. We name this mapping a basic operator $f_1(\cdot,\cdot)$ and obtain the gradient of updated nodal positions with respect to the initial states and rotation angle as:

$$\frac{\partial X_1}{\partial {}^{0}x_j} = \frac{\partial f_1(X_0, \Delta\theta)}{\partial {}^{0}x_j} = \frac{\partial f_1}{\partial X_0}\frac{\partial X_0}{\partial {}^{0}x_j}, \qquad \frac{\partial X_1}{\partial \Delta\theta} = \frac{\partial f_1(X_0, \Delta\theta)}{\partial \Delta\theta} = \frac{\partial f_1}{\partial \Delta\theta}\ . \tag{S11}$$

For example, for all the nodes in Fig. S2b, we have

$$\frac{\partial\, {}^{\mathrm{t}}x_i}{\partial\, {}^{0}x_j} = \delta_{1j} + \left(\delta_{ij} - \delta_{1j}\right)\cos\left(\frac{\Delta\theta}{2}\right),$$

$$\frac{\partial\, {}^{\mathrm{t}}x_i}{\partial \Delta\theta} = -\frac{(1-\delta_{1i})}{2}\left[\left({}^{0}x_i - {}^{0}x_1\right)\sin\left(\frac{\Delta\theta}{2}\right) + \left({}^{0}y_i - {}^{0}y_1\right)\cos\left(\frac{\Delta\theta}{2}\right)\right]. \tag{S12}$$

Based on the updated state, we can proceed to the next panel in the sequence, which is located below the current panel, as shown in Fig. S2c. Since we have obtained $({}^{\mathrm{t}}x_1, {}^{\mathrm{t}}y_1)$ and $({}^{\mathrm{t}}x_2, {}^{\mathrm{t}}y_2)$, the location of node 5 in this panel can be easily solved as

$${}^{\mathrm{t}}x_5 = {}^{\mathrm{t}}x_1, \qquad {}^{\mathrm{t}}y_5 = {}^{\mathrm{t}}y_1 + (C_1 + C_2)\cdot({}^{\mathrm{t}}y_2 - {}^{\mathrm{t}}y_1), \tag{S13}$$

where

$$C_1 = \sqrt{({}^{\mathrm{t}}y_2 - {}^{\mathrm{t}}y_1)^2}, C_2 = \sqrt{({}^0x_5 - {}^0x_2)^2 + ({}^0y_5 - {}^0y_2)^2 - ({}^{\mathrm{t}}x_2 - {}^{\mathrm{t}}x_1)^2}\,. \tag{S14}$$

Similarly, we can consider equation (S13) a transformation mapping $X_2 = f_2(X_1, X_0)$ from the state X_1 to another partially updated state $X_2 = \{({}^{\mathrm{t}}x_i, {}^{\mathrm{t}}y_i)|i = 5\}$. We name this mapping a basic operator $f_2(\cdot,\cdot)$. Therefore, we can now express X_2 as a function of the rotated angle $\Delta\theta$ and initial state X_0 by composing the two basic operators together

$$X_2 = f_2(X_1, X_0) = f_2(f_1(X_0, \Delta\theta), X_0) = X_2(X_0, \Delta\theta). \tag{$S15$}$$

We can obtain the gradient of updated nodal positions with respect to the initial states and rotation angle as:

$$\frac{\partial X_2}{\partial {}^0x_j} = \frac{\partial f_2(X_1, X_0)}{\partial {}^0x_j} = \frac{\partial f_2}{\partial X_1}\frac{\partial X_1}{\partial {}^0x_j} + \frac{\partial f_2}{\partial X_0}\frac{\partial X_0}{\partial {}^0x_j}, \qquad \frac{\partial X_2}{\partial \Delta\theta} = \frac{\partial f_2(X_1, X_0)}{\partial \Delta\theta} = \frac{\partial f_2}{\partial X_1}\frac{\partial X_1}{\partial \Delta\theta}, \tag{S16}$$

in which $\partial f_2/\partial X_1$ and $\partial f_2/\partial X_0$ can be easily obtained from equation (S13) while $\partial X_1/\partial {}^0x_j$ and $\partial X_1/\partial \Delta\theta$ are given in equation (S11).

We can sequentially carry on this nodal location updating process to determine the new configuration for the whole kirigami. Each update involves solving a simple geometric problem, which allows us to easily obtain both the analytical solution of transformation and its gradient. Utilizing the chain rule, we can calculate the gradient of the updated configurations with respect to the initial configuration and the rotation

angle. Given the regularity inherent in the cutting pattern, this sequential process only involves a small set of basic transformations, as shown in Fig. 3e, derived from geometric problem prototypes (illustrated in Fig. 3b and Fig. S2). Consequently, in practical implementation, it is only necessary to define an analytical subfunction for each basic transformation. The analytical expression for the entire updated configuration $X = \chi(X_0, \Delta\theta)$ can then be easily obtained by composing these basic subfunctions in a certain sequence. The gradient of X can be obtained by using the chain rule that multiplies gradients of those basic operators in inverse order.

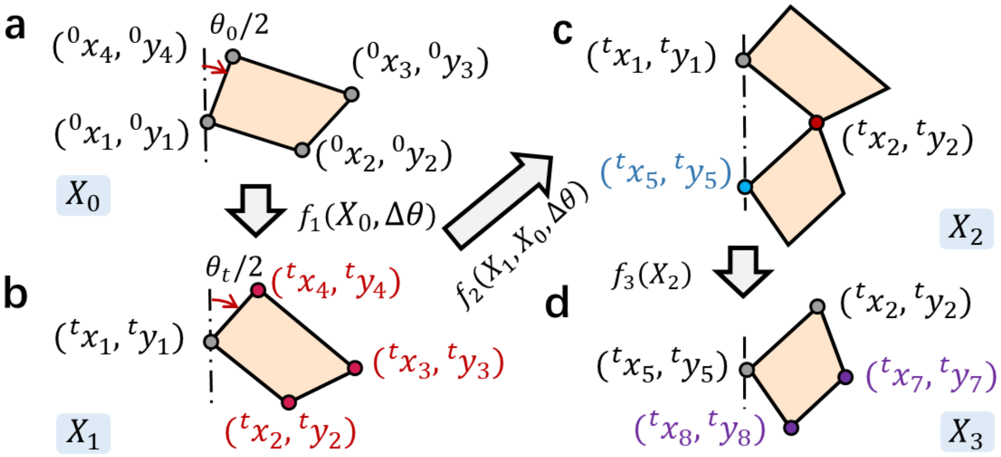

Supplementary Figure 2. Exemplified transformation series in the differentiable kinematic analysis to update the nodes of panels in the center two columns, corresponding to Fig. 3a~c. **a** The initial state of the first-panel **b** Transforming the first panel to the new position corresponding to the red arrows in Fig. 3c. The coordinates of red nodes are updated based on the preceding known nodes colored in grey. It corresponds to the geometrical problem prototype marked by red shaded regions in Fig. 3b. **c** Transforming node 5 in the second panel to its new state, corresponding to the blue arrows Fig.3c. The geometrical problem prototype corresponds to the blue shaded regions in Fig. 3b. **d** Transforming the remaining nodes of the second panel to the new state corresponding to the purple arrows in Fig.3c. The geometrical problem prototype corresponds to the purple shaded regions in Fig. 3b.

Supplementary Note 3. Finite element analysis

To validate the proposed energy-based simulation method, a user-defined element subroutine in the commercial software ABAQUS was utilized. This subroutine is based on the theoretical framework developed by Zhao et al.[3], which incorporates the magneto-elastic behavior of hard-magnetic soft active materials. It combines the strain energy of the soft matrix with the magnetic potential of the embedded hard-magnetic particles. The magnetic moment density, shear modulus, and Poisson's ratio are set to be 70 kA m^{-1}, 300 kPa, and 0.495 respectively, which matches the materials used in physical experiments. The simulated configurations in Fig. S5a and Fig. S6a-e are obtained by this user-defined element subroutine. Considering the symmetry of the structure, we performed symmetric analysis on the right half of the kirigami, in which the $7 \times 7 \times 0.08$ cm structure is meshed into 4292 elements with one element along the thickness direction.

Supplementary Note 4. Hyper-elastic hinge model

Fig. S3a-b illustrates the introduction of gaps between panels to realize the desired cutting patterns in the 3D printing process. In the printed sample, each pair of panels is connected by a cuboid hinge with thickness b, height h, and length l (Fig. S3c). Since we observed that the majority of the deformation occurs in the hinges through rotation while the panels undergo negligible deformation, we assume the panels to be rigid and connected by rotational springs to create a simplified model for the kirigami structure (Fig. S3d). In most existing kirigami designs, linear elasticity is assumed for the hinge materials, resulting in a linear spring model to describe the rotational moment and elastic energy. However, this linearity assumption is not valid in our case due to the significant deformation of the hyperelastic polymer. Therefore, we developed a nonlinear spring model that describes the hinge deformation using a hyperelastic beam model (Fig. S3e)[4]. The beam model incorporates the Euler-Bernoulli kinematic hypothesis, incompressibility assumption, and the thickness-stretching effect, which is particularly important for incompressible or nearly incompressible soft structures experiencing large strains. The neo-Hookean model is used as the constitutive model for the incompressible/near-incompressible materials with shear modulus G. By

applying the theory of finite elasticity and standard variational methods, analytical formulas can be derived to describe the moment magnitude τ induced by pure bending with angle β as

$$\tau(\beta) = -\frac{Gbl^2}{2\beta^2} ln\left(\sqrt{1+\frac{\beta^2 h^2}{l^2}} + \frac{\beta h}{l}\right) + \frac{Gbl}{2\beta}\sqrt{1+\frac{\beta^2 h^2}{l^2}}. \tag{S17}$$

To validate this nonlinear spring model, we use it to calculate the moments induced by different bending angles for a typical hinge used in all our kirigami designs ($b = 0.8$ mm, $h = 1.2$ mm, $l = 1.6$ mm, $G = 300$ kPa). We compare it with FEA using C3D8R elements detailed in Supplementary Note S3 and the commonly used linear beam model. The linear beam model is given as

$$\tau_l(\beta) = \frac{Gbh^2}{3l}\beta. \tag{S18}$$

The comparative results are presented in Fig. S3f. It can be observed that the commonly used linear model matches with FEA only for small bending angles ($\beta < 30°$), but shows significant discrepancies for large bending angles. In contrast, our nonlinear spring model closely approximates the FEA results even for large bending angles, demonstrating its validity and improved accuracy in capturing the behavior of the kirigami structure. Therefore, we chose to use the nonlinear model to simulate and optimize the kirigami patterns in this study.

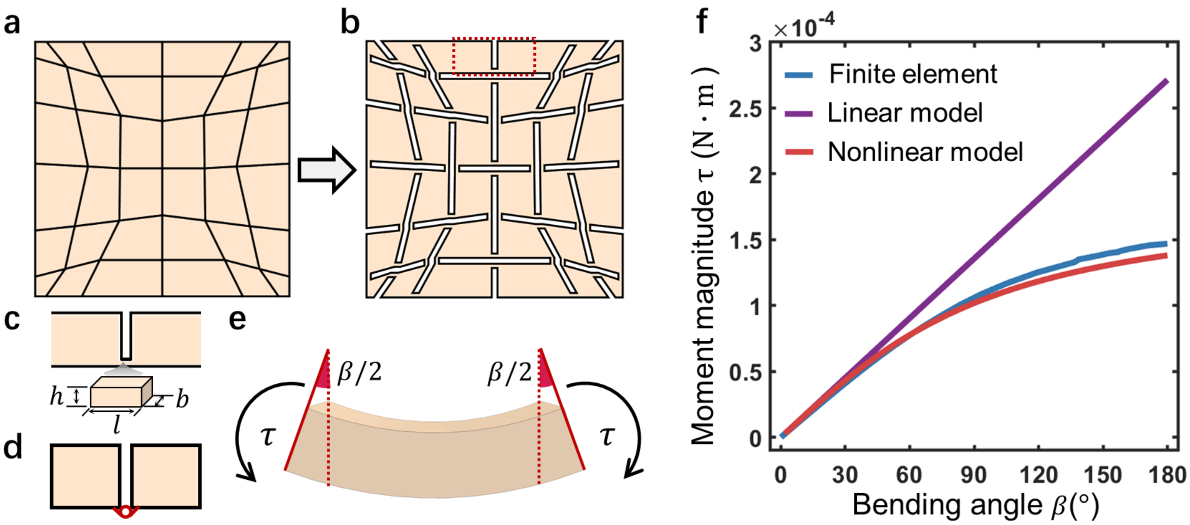

Supplementary Figure 3. Illustration of the hinge model. **a** An exemplified kirigami cutting pattern and its **b** printable structure by replacing the cutting with gaps and connecting panels with hinges. **c** Enlarged hinge region corresponding to the dashed rectangular region in Fig. S3b. The dimensions of the hinge are marked in a zoom-in inset at the bottom. **d** Equivalent nonlinear torsional spring model. The panels are assumed to be rigid and connected by a nonlinear torsional spring. **e** Hyperplastic beam model as the nonlinear torsional spring. **f** Comparative results between FEA, linear beam model, and the proposed hyper-elastic beam model. Source data are provided as a Source Data file.

Supplementary Note 5. Differentiable magneto-elastic energy model and energy-based simulation

In the analysis of kirigami structures, determining the physical equilibrium by analyzing the interactive forces between panels can be a complex task due to the intricate nature of their interactions. As an alternative approach, we assess the physical equilibrium by examining whether the total energy of the kirigami structure reaches a minimum. To integrate this energy-based analysis into the optimization, we introduce a differentiable energy model that combines analytical elastic and magnetic energies. Herein, we focus on describing the energy of a kirigami design with a deployed angle θ, nodal coordinates $X = \{(x_i, y_i)\}$ and magnetization orientation $\Psi = \{\mathbf{e}_i\}$ of each panel.

S5.1 Elastic energy model

From the nonlinear spring model introduced earlier, we can have the elastic energy of a hinge associated with the bending angle β as

$$\pi_{\mathrm{e}}(\beta) = \int \tau(\beta) d\beta\,. \tag{S19}$$

In our implementation, the above energy value can be efficiently obtained by using the built-in symbolic integration function in MATLAB. Its gradient with respect to the bending angle is simply the analytical formulation in equation (S17),

$$\frac{\partial \pi_{\mathrm{e}}(\beta)}{\partial \beta} = \tau(\beta) = -\frac{Gbl^2}{2\beta^2} ln\left(\sqrt{1+\frac{\beta^2 h^2}{l^2}} + \frac{\beta h}{l}\right) + \frac{Gbl}{2\beta}\sqrt{1+\frac{\beta^2 h^2}{l^2}}\,. \tag{S20}$$

We assume that, kirigami in the rest state, i.e., no deformation in the hinges, has a deployed angle θ_r. Based on the aforementioned sequential kinematic analysis, we can derive an analytical expression for nodal coordinates at the rest state $X_{\mathrm{r}} = \chi(X, \Delta\theta_{\mathrm{r}}) = \{({}^{\mathrm{r}}x_i, {}^{\mathrm{r}}y_i)\}$, where $\Delta\theta_{\mathrm{r}} = \theta_{\mathrm{r}} - \theta$ represents the difference in angles between the rest state and the current state. Using the nodal locations at the current and rest states, i.e., X and X_{r}, we can easily obtain an analytical expression for the bending angle β_i for every hinge V_i, i.e., $\beta_i(X, X_{\mathrm{r}})$. Therefore, the total elastic energy of the kirigami structure at a given deployed angle θ can be calculated by summing the elastic energies of all the hinges together,

$$\Pi_{\mathrm{e}} = \sum_{i=1}^{n_{\mathrm{h}}} \pi_{\mathrm{e}}\big(\beta_i(X, X_{\mathrm{r}})\big)\,, \tag{S21}$$

where n_h is the total number of hinges in the kirigami, β_i is the bending angle of the ith hinge and θ_r is considered a constant parameter for a given kirigami design.

S5.2 Modified elastic energy model

In the previous elastic model, the assumption was made that the panels in the kirigami structure are rigid and do not undergo any deformation. As shown in Fig. S4a, this means that the rotated angle β_i between the two neighboring panels is exactly the bending angle of the hinges. However, in reality, the regions connected to the hinges also experience small deformations, as shown in Fig. S4b. As a result, the rotated angle β_i will be slightly larger than the real bending angle of the hinge $\tilde{\beta}_i$ in achieving a specific configuration. To account for this, we propose modifying the bending angle of the hinges to compensate for the overestimation caused by the rigid panel assumption. This modification is based on the simplified geometrical descriptions in Fig. S4c.

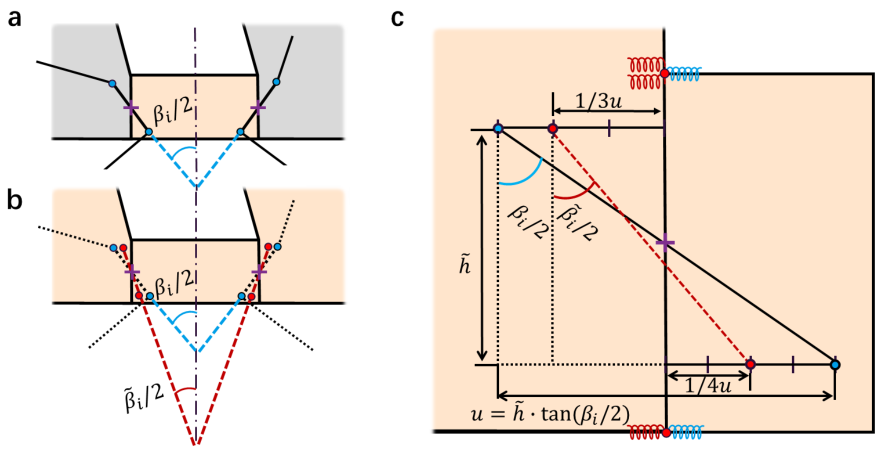

Supplementary Figure 4. Illustration of the modified bending angle. **a** Bending angle with rigid panel assumption. Grey and orange regions represent panels and the hinge. The blue dots mark the new positions of the four ends in the hinge after the bending. **b** Real bending angle with local deformation in the panel. The red dots mark the new positions of the four ends in the hinge after the bending. **c** Simplified geometry model to derive the modified bending angle.

For a given rotation angle β_i between two connected panels, we assume that both panels rotate by $\beta_i/2$, allowing us to focus on just one of them. To simplify the analysis, we divide the deformation process associated with the rotation into two stages. In the first stage, we freeze the entire panel and rotate it by $\beta_i/2$. This causes the cross-section of the hinge directly connected to the rigid panel to tilt by $\beta_i/2$, as marked by the black dashed lines in Fig. S4c. In the second stage, we unfreeze the panel and allow it to deform. The upper half of the hinge, being stretched in the first stage, will thus shrink back slightly in this stage. As a simplified assumption, we assume that the upper end retains its vertical position and only shrinks horizontally, as shown in the upper two colored dots in Fig. S4c. When the upper end of the hinge undergoes a unit shrinking, there is an increase in reaction forces on the panel side and a decrease on the hinge side. The increase in reaction forces on the panel side is twice as large as the decrease on the hinge side because the panel side has an extra half-plane bonding with the upper end. This difference in reaction forces causes the upper end to shrink back to approximately two-thirds of its original elongation distance, reaching a new equilibrium state. Following a similar procedure, we can also conclude that the lower end will retain half of its compressed distance. The bending angle of the hinge can then be modified as

$$\tilde{\beta}_i = 2 \cdot \arctan\left[\frac{7}{12}\tan\left(\frac{\beta_i}{2}\right)\right]. \tag{S22}$$

After replacing β_i with $\tilde{\beta}_i$ in equation (S21), we obtain the modified elastic energy model as

$$\Pi_\mathrm{e} = \sum_{i=1}^{n_\mathrm{h}} \pi_\mathrm{e}(\tilde{\beta}_i), \tag{S23}$$

S5.3 Magnetic energy model

For each panel, we can calculate its area A_i based on the locations of its four nodes analytically. When exposed to an external stimulus, the corresponding magnetic potential energy of the ith panel is given as

$$\pi_{\mathrm{m}} = -bMA_i\mathbf{e}_i \cdot \mathbf{B}, \tag{S24}$$

where M and $\mathbf{e}_i$ are the magnitude and orientation of magnetization for embedded particles, respectively, and $\mathbf{B}$ is the magnetic flux density vector of the uniform external magnetic field, b is the thickness of the kirigami. It can be observed from equation (S24) that, as the magnetization orientation rotates from the direction of the external field to the opposite, the magnetic potential will increase. The induced magnetic torque can be given as

$$\tau_{\mathrm{m}} = bMA_i\mathbf{e}_i \times \mathbf{B}. \tag{S25}$$

Unlike the energy potential, the magnitude of the induced magnetic torque will peak when the magnetization vector is perpendicular to the external field and will become zero when aligned in the same line with the external field. The total magnetic energy of the kirigami structure at a given deployed angle θ can be calculated by summing the magnetic potentials of all the panels together,

$$\Pi_{\mathrm{m}} = \sum_{i=1}^{n_{\mathrm{p}}} -bMA_i\mathbf{e}_i \cdot \mathbf{B}\,, \tag{S26}$$

where n_{p} is the total number of panels in the kirigami.

S5.4 Energy-based simulation

The actuated configuration of kirigami should be in a state of equilibrium, characterized by the lowest possible total energy under a given magnetic field. Therefore, with the differentiable elastic and magnetic energy models introduced, we can simulate the actuated state by framing it as an energy minimization

problem. Specifically, suppose all the kirigami design parameters, i.e., nodal coordinates $X = \{(x_i, y_i)\}$ and magnetization orientation $\Psi = \{\mathbf{e}_i\}$, are given in a state with a deployed angle $\theta(X)$. We also know that the rest state of the kirigami corresponds to a deployed angle θ_r. Then, for any given deployed angle $\theta_\mathrm{t} = \theta + \Delta\theta_\mathrm{t}$, we can use the sequential kinematic analysis to obtain the corresponding nodal coordinates $X_\mathrm{t} = \{({}^\mathrm{t}x_i, {}^t y_i)\} = \chi(X, \Delta\theta_\mathrm{t})$. The magnetization orientation can be updated based on the rotation of each panel, which is also an analytical function $\Psi_\mathrm{t} = \{{}^\mathrm{t}\mathbf{e}_i\} = \Psi_\mathrm{t}(X, \Psi, X_\mathrm{t})$. The magnetic potential can be expressed as

$$\Pi_\mathrm{m} = \sum_{i=1}^{n_\mathrm{p}} -bMA_i \cdot {}^\mathrm{t}\mathbf{e}_i \cdot \mathbf{B}. \tag{S27}$$

Note that the area of each panel remains the same and independent from the deployment process. The total energy Π_t can be derived from equations (S23) and (S27) as

$$\Pi_\mathrm{t} = \Pi_\mathrm{e} + \Pi_\mathrm{m} = \sum_{i=1}^{n_\mathrm{h}} \pi_\mathrm{e}(\tilde{\beta}_i) + \sum_{i=1}^{n_\mathrm{p}} -bMA_i \cdot {}^\mathrm{t}\mathbf{e}_i \cdot \mathbf{B}. \tag{S28}$$

Given any kirigami design X and Ψ, we can find the corresponding deployed angle at the equilibrium state under external actuation **B** by minimizing the total energy

$$\tilde{\theta} = \arg\min_{\theta_\mathrm{t}} \Pi_\mathrm{t}. \tag{S29}$$

The corresponding actuated configuration can then be obtained from the kinematic analysis as

$$\tilde{X} = \chi(X, \tilde{\theta} - \theta). \tag{S30}$$

Since every term in the total energy is differentiable, we can easily derive the gradient of the total energy as

$$\frac{\partial \Pi_{\mathrm{t}}}{\partial \theta_{\mathrm{t}}}=\frac{\partial \Pi_{\mathrm{e}}}{\partial \theta_{\mathrm{t}}}+\frac{\partial \Pi_{\mathrm{m}}}{\partial \theta_{\mathrm{t}}}=\sum_{i=1}^{n_{\mathrm{h}}}\frac{\partial \pi_{\mathrm{e}}(\tilde{\beta}_i)}{\partial \theta_{\mathrm{t}}}+\sum_{i=1}^{n_{\mathrm{p}}}-MA_i\frac{\partial\, {}^{\mathrm{t}}\mathbf{e}_i}{\partial \theta_{\mathrm{t}}}\cdot\mathbf{B}. \tag{S31}$$

In the first term, we have

$$\frac{\partial \pi_{\mathrm{e}}(\tilde{\beta}_i)}{\partial \theta_{\mathrm{t}}}=\frac{\partial \pi_{\mathrm{e}}(\tilde{\beta}_i)}{\partial \tilde{\beta}_i}\frac{\partial \tilde{\beta}_i}{\partial \beta_i}\frac{\partial \beta_i(X_{\mathrm{t}},X_{\mathrm{r}})}{\partial X_{\mathrm{t}}}\frac{\partial X_{\mathrm{t}}}{\partial \Delta\theta_{\mathrm{t}}}\frac{\partial \Delta\theta_{\mathrm{t}}}{\partial \theta_{\mathrm{t}}}=\tau(\tilde{\beta}_i)\frac{\partial \tilde{\beta}_i}{\partial \beta_i}\frac{\partial \beta_i(X_{\mathrm{t}},X_{\mathrm{r}})}{\partial X_{\mathrm{t}}}\frac{\partial \chi(X,\Delta\theta_{\mathrm{t}})}{\partial \Delta\theta_{\mathrm{t}}}, \tag{S32}$$

where $\tau(\tilde{\beta}_i)$ is given in equation (S17), $\frac{\partial \beta_i(X_{\mathrm{t}},X_{\mathrm{r}})}{\partial X_{\mathrm{t}}}$ and $\frac{\partial \chi(X,\Delta\theta_{\mathrm{t}})}{\partial \Delta\theta_{\mathrm{t}}}$ can be derived analytically from the differentiable kinematic analysis, and $\frac{\partial \tilde{\beta}_i}{\partial \beta_i}$ can be obtained from equation (S22) as

$$\frac{\partial \tilde{\beta}_i}{\partial \beta_i}=\frac{\frac{7}{12}\left[\tan\left(\frac{\beta_i}{2}\right)^2+1\right]}{\frac{49}{144}\tan\left(\frac{\beta_i}{2}\right)^2+1}. \tag{S33}$$

In the second term of equation (S31), we have

$$\frac{\partial\, {}^{\mathrm{t}}\mathbf{e}_i}{\partial \theta_{\mathrm{t}}}=\frac{\partial\, {}^{\mathrm{t}}\mathbf{e}_i}{\partial X_{\mathrm{t}}}\frac{\partial \chi(X,\Delta\theta_{\mathrm{t}})}{\partial \Delta\theta_{\mathrm{t}}}\frac{\partial \Delta\theta_{\mathrm{t}}}{\partial \theta_{\mathrm{t}}}=\frac{\partial\, {}^{\mathrm{t}}\mathbf{e}_i}{\partial X_{\mathrm{t}}}\frac{\partial \chi(X,\Delta\theta_{\mathrm{t}})}{\partial \Delta\theta_{\mathrm{t}}}, \tag{S34}$$

which can also be obtained analytically via the differentiable kinematic analysis. As a result, the energy minimization problem defined in equation (S29) becomes a simple optimization problem with only one optimization variables θ_{t} and analytical gradient in equation (S31), which can be efficiently solved by any

gradient-based optimizer. It should be pointed out that, since both the elastic energy and magnetic potential have a linear relation with the thickness of kirigami (hinge), the solution of the stable deployed angle θ_t stay the same for different thickness values. In this study, we use the built-in function *fmincon* in MATLAB to solve it with θ as the initial guess. As a validation, we compare the actuated configurations for the same kirigami obtained by FEA and the proposed energy minimization method in Fig. S5. We also include the simulated results by using the original elastic model (equation (S21)) in the energy optimization, assuming perfectly rigid panels. In the comparative case, the magnetic moment density, shear modulus, and Poisson's ratio are set to be 70 kA m-1, 300 kPa, and 0.495 respectively, which matches the materials used in all the numerical designs and physical experiments. The magnetic field aligns along the positive y-axis with the magnitude s $B = 30$ mT. From the results, we can see that energy minimization with the original elastic model overestimates the elastic energy, which leads to an actuated configuration with a much smaller deployed angle (Fig. S5b) than the ground truth obtained by FEA (Fig. S5a). In contrast, with the modified elastic model, the energy minimization gives a very accurate simulation (Fig. S5c) and matches well with the FEA.

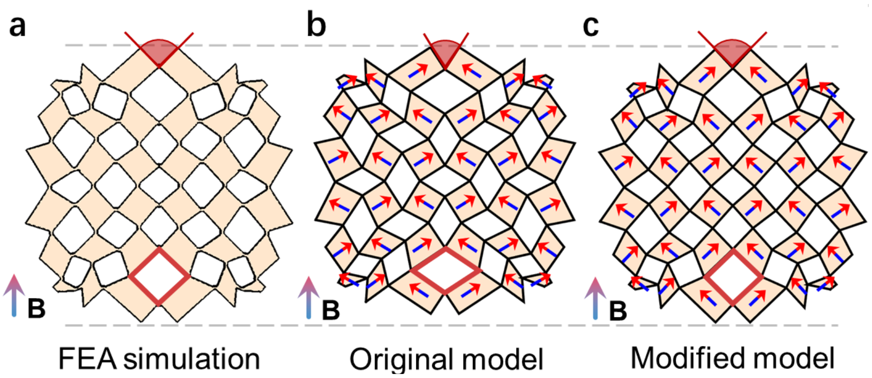

Supplementary Figure 5. Simulated deployed states of the kirigami by **a** FEA, **b** original energy-based model with rigid-panel assumption, and **c** energy-based model with modified bending angles.

We further compare the simulated results obtained by FEA and our energy minimization methods under different levels of magnetic fields, as shown in Fig. S6. It is evident that the configurations obtained from

the two methods match well in all cases. It should be noted that a single FEA simulation takes around ten minutes and might even not converge under strong magnetic stimuli. In contrast, the energy-based simulation only takes seconds to finish with a more stable solving process.

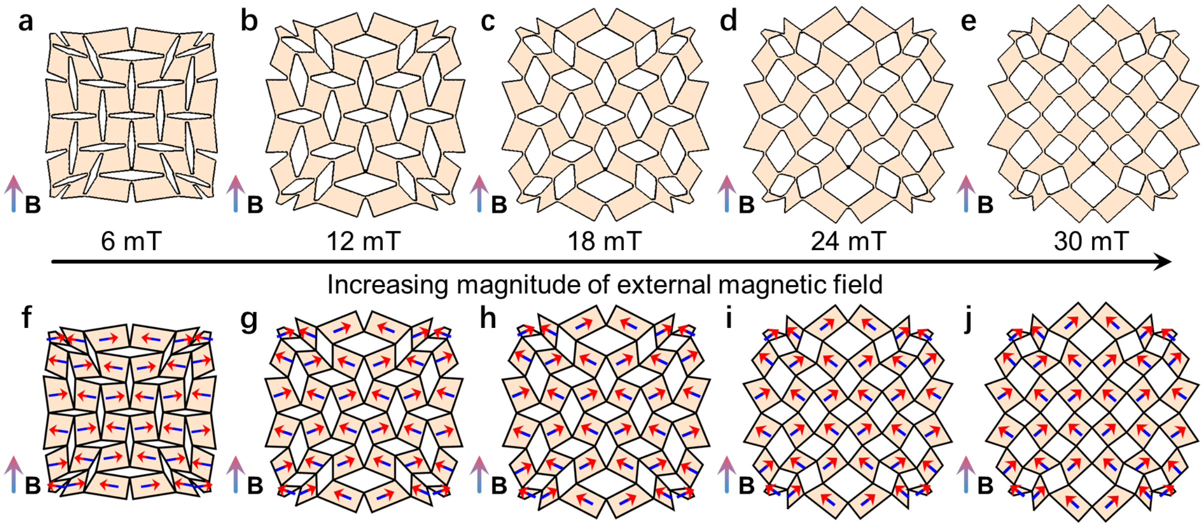

Supplementary Figure 6. Simulated deployed states of the same kirigami as in Fig. S5 but under different magnitudes of stimuli, obtained via **a** to **e** FEA and **f** to **j** energy-based model with modified bending angles.

Supplementary Note 6. Physics-aware kirigami optimization

S6.1 Optimization with one compact state

As illustrated in the main text, the design variables for kirigami include the nodal position of nodes $X = \{(x_i, y_i)\}$ and the vertical component of the magnetization orientation d_i in each panel. The magnetization orientation can be described as $\mathbf{e}_i = \left(\gamma_i\sqrt{1-d_i^2}, d_i\right)$, where γ_i corresponds to the sign of horizontal magnetization components marked in Fig. 2a. The design of a shape-morphing kirigami is essentially to identify the nodal position of nodes $X = \{(x_i, y_i)\}$ and magnetization orientation $\Psi = \{\mathbf{e}_i\}$ so that they can meet all the geometrical requirements and ensure a physical equilibrium state. We can use any of the requirements, either geometrical or physical, as the design objective and leave others as constraints based

on different design scenarios. Herein, we choose to incorporate physical equilibrium requirements into the design objective while using all the geometrical requirements as constraints for consistency.

A naïve way to consider physical equilibrium in the objective is to use the discrepancy between the designed deployed angle θ and the simulated real deployed angle $\tilde{\theta}$ in equation (S29) as the objective function for minimization. However, the search for $\tilde{\theta}$ itself requires an iterative optimization process, making this naïve objective non-differentiable and impetus the use of effective gradient-based methods. Another possible choice of the physical objective function is to directly search for X and Ψ which minimizes the total energy function in equation (S28). Nevertheless, the objective function significantly shrinks the optimal design sets, because its optimality criteria are much stricter than the physical equilibrium requirements. Specifically, the physical equilibrium only requires θ to have the lowest energy state for a ***given*** X and Ψ, whereas the energy-minimization-based objective aims for the lowest energy for ***all possible*** X and Ψ. As a result, this objective function will make the search for a physically stable kirigami design more complicated than necessary.

In contrast, we propose to use the squared gradient of the total energy with respect to the deployed angle as the minimization objective, i.e., $\min_{x_i,y_i,d_i}\left(\frac{\partial \Pi_{\mathrm{t}}}{\partial \theta}\right)^2$. The rationale behind this is that in a physical equilibrium state, the total energy of the system exhibits zero gradients with respect to the deployed angle. This means that any small perturbation in the configuration will result in an increase in the total energy. Therefore, the system is at a stable point where it is energetically favorable and resistant to perturbations. Since the gradient values around the equilibrium state will be small in scale, we take its logarithm as the objective for better optimization performance

$$\min_{x_i,y_i,d_i} \mathcal{F} = \ln\left[\left(\frac{\partial \Pi_{\mathrm{t}}}{\partial \theta}\right)^2 + \delta\right], \tag{S35}$$

where $\delta = 2 \times 10^{-16}$ is a small constant to avoid singularity issues. The gradient of this objective function is given as

$$\frac{\partial \mathcal{F}}{\partial x_i} = 2\left[\left(\frac{\partial \Pi_{\mathrm{t}}}{\partial \theta}\right)^2 + \delta\right]^{-1} \cdot \frac{\partial \Pi_{\mathrm{t}}}{\partial \theta} \cdot \frac{\partial^2 \Pi_{\mathrm{t}}}{\partial \theta\, \partial X} \cdot \frac{\partial X}{\partial x_i}, \tag{S36}$$

$$\frac{\partial \mathcal{F}}{\partial y_i} = 2\left[\left(\frac{\partial \Pi_{\mathrm{t}}}{\partial \theta}\right)^2 + \delta\right]^{-1} \cdot \frac{\partial \Pi_{\mathrm{t}}}{\partial \theta} \cdot \frac{\partial^2 \Pi_{\mathrm{t}}}{\partial \theta\, \partial X} \cdot \frac{\partial X}{\partial y_i}, \tag{S37}$$

$$\frac{\partial \mathcal{F}}{\partial d_i} = 2\left[\left(\frac{\partial \Pi_{\mathrm{t}}}{\partial \theta}\right)^2 + \delta\right]^{-1} \cdot \frac{\partial \Pi_{\mathrm{t}}}{\partial \theta} \cdot \frac{\partial^2 \Pi_{\mathrm{t}}}{\partial \theta\, \partial \mathbf{e}_i} \cdot \frac{\partial \mathbf{e}_i}{\partial d_i}. \tag{S38}$$

It should be noted that, when the compact state is considered as the rest state, i.e., $\theta_{\mathrm{r}} = 0$ and $\Delta\theta_{\mathrm{r}} = \theta_{\mathrm{r}} - \theta = -\theta$, neighboring panels will have the cutting between them compact in this rest state. As a result, the bending angle of each hinge is only dependent on the configuration of the current state, i.e., $\beta_i = \beta_i(X)$. The objective function in equation (S35) thus only depends on the design at the deployed state and does not require obtaining the detailed configurations at the compact state. With the differentiable kinematic and energy models, analytical expressions for equations (S36)-(S38) can be readily obtained. Consequently, the minimization problem, incorporating the geometric constraints mentioned, can be efficiently solved using gradient-based nonlinear optimization methods. In this study, we have chosen to employ sequential quadratic programming (SQP) as the optimization method for energy minimization[5]. This decision is justified by the widespread adoption of SQP in handling nonlinear, constrained optimization problems, which aligns well with the nature of our problem. Additionally, SQP is recognized for its robustness and efficiency, making it a suitable choice for addressing complex optimization challenges. The implementation of SQP in this study is achieved through the utilization of the built-in function *fmincon* in MATLAB.

S6.2 Two-way contractible design

As shown in Fig. 6a, two-way contractible design involves three different states of the kirigami, i.e., a negative state S_-, a zero state S_0, and a positive state S_+. The negative and positive states are two compact states associated with the same deployed kirigami in the zero state. In the two-way design, we assume the zero state is a rest state without any elastic deformation or external excitation. The goal is to design a kirigami in the zero state that can transform into negative and positive states given stimuli $-\mathbf{B} = -B\boldsymbol{e}_z$ and $\mathbf{B} = B\boldsymbol{e}_z$, respectively. To achieve this, both negative and positive states should be in physical equilibrium under their respective excitations. For the ease of later illustration, we denote the energy gradients in equation (S31) evaluated at the negative state S_-, with the rest state S_0 and excitation $-\mathbf{B}$, as $\mathcal{g}(S_-|S_0, -\mathbf{B})$. Similarly, the energy gradients evaluated at the positive state S_+ with rest state S_0 and excitation $\mathbf{B}$ is denoted as $\mathcal{g}(S_+|S_0, \mathbf{B})$. The physical equilibrium in the two-way contractible design thus implies

$$\mathcal{g}(S_-|S_0, -\mathbf{B}) = 0, \mathcal{g}(S_+|S_0, \mathbf{B}) = 0. \tag{S39}$$

The objective function can then be modified by aggregating the two zero energy gradient requirements as

$$\min_{x_i, y_i, d_i} \mathcal{F} = \ln[\mathcal{g}(S_-|S_0, -\mathbf{B})^2 + \mathcal{g}(S_+|S_0, \mathbf{B})^2 + \delta]\,. \tag{S40}$$

This two-way contractible objective function needs to be evaluated at both compact states, but the design variables are defined at the deployed space. It is different from the design with only one compact state (equation (S35)), in which the objective and design variables are both defined in the deployed state. Although we can express the configurations of the two compact states as a function of the configuration in the deployed state, the resulting expression will be extremely complicated and highly nonlinear to optimize. To mitigate this difficulty, we propose a two-stage design method by extending our previous optimization approach.

In the first stage, we introduce a dual problem for each of the compact states (Fig. S7). For the dual problem associated with the negative compact state, we consider the compact kirigami S_- as the rest state. We reverse the magnetic field orientation to be positive $\mathbf{B}$ and then seek to design a deployed state S_0 with minimal total energy, which can be expressed as

$$\min_{x_i, y_i, d_i} \bar{\mathcal{F}}_- = \ln[\mathcal{g}(S_0|S_-, \mathbf{B})^2]\,. \tag{S41}$$

Similarly, we obtain the dual problem for the positive compact state as

$$\min_{x_i, y_i, d_i} \bar{\mathcal{F}}_+ = \ln[\mathcal{g}(S_0|S_+, -\mathbf{B})^2]\,. \tag{S42}$$

By aggregating equations (S41) and (S42), the objective function for the first stage can be given as

$$\min_{x_i, y_i, d_i} \bar{\mathcal{F}} = \ln[\mathcal{g}(S_0|S_-, \mathbf{B})^2 + \mathcal{g}(S_0|S_+, -\mathbf{B})^2 + \delta]\,. \tag{S43}$$

Comparing the first-stage objective in equation (S43) with the original objective in equation (S40), it can be noted that we simply switch the role of S_- and S in the energy gradient evaluation and flip the direction of stimuli. As a result, the objective and the design variables are again both defined in the same deployed state. It is essentially the same as the optimization problem in equation (S36) with only one compact state, which can be efficiently solved using the SQP method. We denote the solution as $\{\tilde{x}_i, \tilde{y}_i, \tilde{d}_i\}$, corresponding to a deployed angle $\tilde{\theta}$. In Fig. S7, we show the first-stage design results for circular deployed shapes. From the energy analysis in Fig. S7h-i, we can observe that the same deployed design becomes the energy minimum for both dual problems of negative and positive states. Specifically, in the dual problem of the

negative state, it means the deployed state S_0 have smaller energy than the negative compact state S_- under excitation $\mathbf{B}$ when S_- is considered as the rest state:

$$\Pi_{\mathrm{t}}(S_0|S_-,\mathbf{B}) = \Pi_{\mathrm{e}}(S_0|S_-) + \Pi_{\mathrm{m}}(S_0|\mathbf{B}) \leq \Pi_{\mathrm{t}}(S_-|S_-,\mathbf{B}) = 0 + \Pi_{\mathrm{m}}(S_-|\mathbf{B}), \tag{S44}$$

in which $\Pi_{\mathrm{e}}(S_0|S_-)$ represents elastic energy of S_0 with S_- as the rest state, $\Pi_{\mathrm{m}}(S_0|\mathbf{B})$ and $\Pi_{\mathrm{m}}(S_-|\mathbf{B})$ represent the magnetic potential energies of S_0 and S_- given $\mathbf{B}$, respectively. From equation (S44), we can further obtain

$$\Pi_{\mathrm{e}}(S_0|S_-) - \Pi_{\mathrm{m}}(S_-|\mathbf{B}) \leq -\Pi_{\mathrm{m}}(S_0|\mathbf{B}). \tag{S45}$$

Since elastic energy stays the same when we switch the role of active and rest states, we have $\Pi_{\mathrm{e}}(S_0|S_-) = \Pi_{\mathrm{e}}(S_-|S_0)$. Meanwhile, the magnetic potential only changes the sign when we fix the configuration but reverse the orientation of the external field, i.e., $-\Pi_{\mathrm{m}}(S_-|\mathbf{B}) = \Pi_{\mathrm{m}}(S_-|-\mathbf{B})$ and $-\Pi_{\mathrm{m}}(S_0|\mathbf{B}) = \Pi_{\mathrm{m}}(S_0|-\mathbf{B})$. Therefore, we can derive from equation (S45) that

$$\Pi_{\mathrm{t}}(S_-|S_0,-\mathbf{B}) = \Pi_{\mathrm{e}}(S_-|S_0) + \Pi_{\mathrm{m}}(S_-|-\mathbf{B}) \leq \Pi_{\mathrm{m}}(S_0|-\mathbf{B}) = \Pi_{\mathrm{t}}(S_0|S_0,-\mathbf{B}). \tag{S46}$$

Similarly, from the dual problem of the positive state, we have

$$\Pi_{\mathrm{t}}(S_+|S_0,\mathbf{B}) = \Pi_{\mathrm{e}}(S_+|S_0) + \Pi_{\mathrm{m}}(S_+|\mathbf{B}) \leq \Pi_{\mathrm{m}}(S_0|\mathbf{B}) = \Pi_{\mathrm{t}}(S_0|S_0,\mathbf{B}). \tag{S47}$$

Equations (S46) and (S47) indicate that the design obtained from the dual problems in the first stage will have higher energy at the zero state than both negative and positive states in the original problem. This can also be observed in the energy analysis of Fig. S8d. While it does not guarantee that the two compact states

are the energy minimum in the original problem, they tend to be close to the minimum due to the symmetry and convexity of the energy functions. As depicted in Fig. S8a-c, the first stage design successfully transforms into a compact pattern in the negative state and achieves a nearly compact pattern in the positive state.

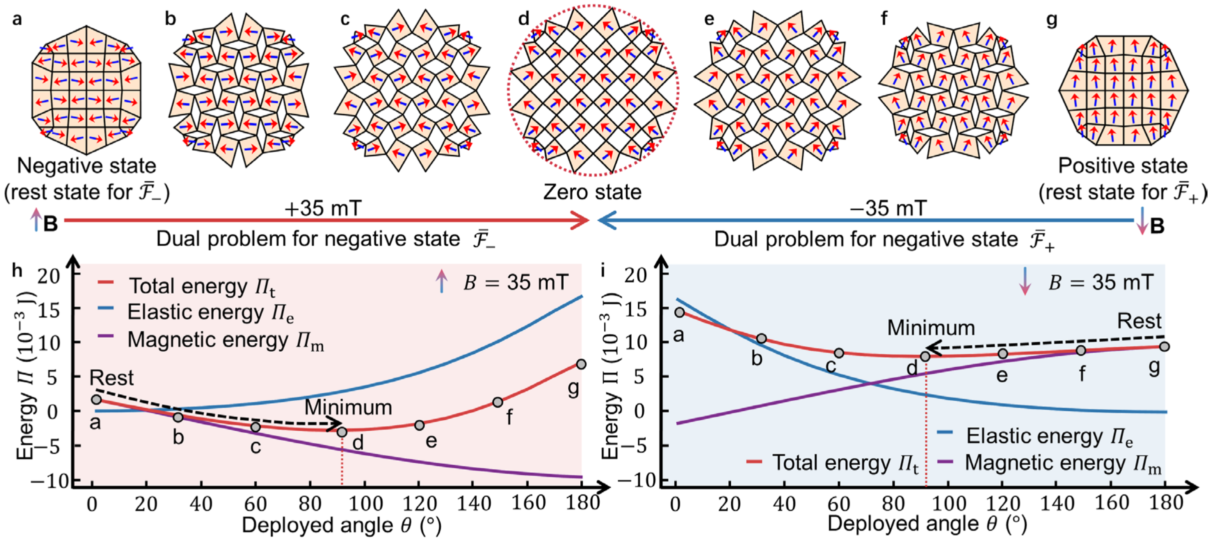

Supplementary Figure 7. First-stage design results. **a-g** Configurations corresponding to different deployed angles. The red and blue arrows show the deployed processes for the dual problems of negative and positive states, respectively. **h** Energy analysis of the design under constant positive excitation (+35 mT) as in the dual problem for the negative state. The grey dots correspond to configurations with the same alphabetic label in the above sub-figures. Source data are provided as a Source Data file. **i** Energy analysis of the design under constant negative excitation (-35 mT) as in the dual problem for the positive state. Source data are provided as a Source Data file.

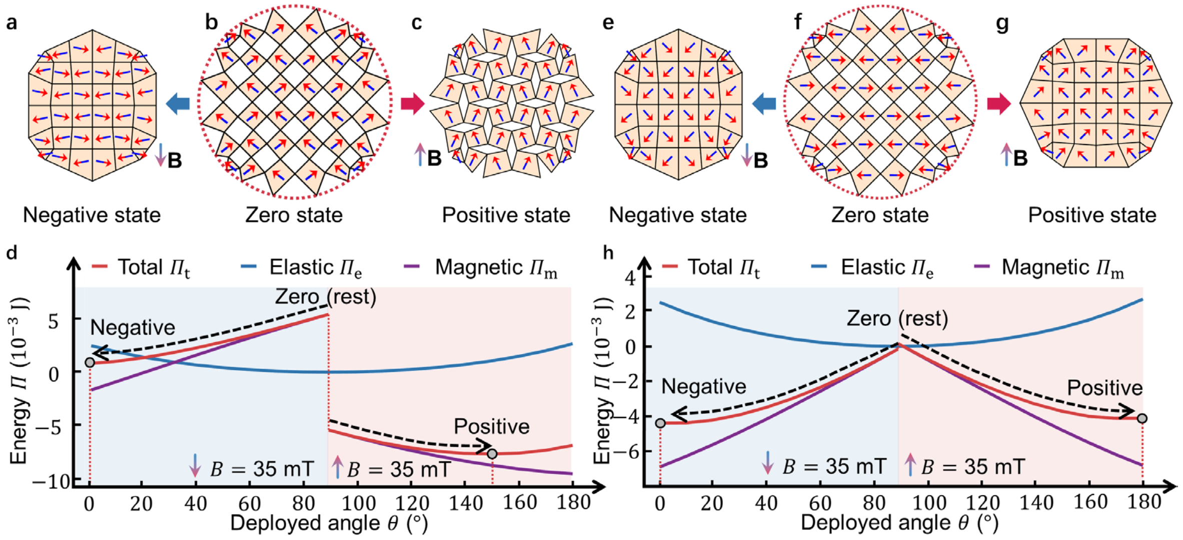

Supplementary Figure 8. Second-stage design results. **a**, b, and **c** show simulated configurations of designed kirigami obtained from the first-stage optimization in Fig. S7, under negative, zero, and positive external magnetic excitations in the original problem, respectively. **d** Energy analysis for the kirigami in Fig. S8b with the setting the same as in Fig. 6c. Regions shaded in red correspond to analysis under constant positive (+35 mT) and constant negative (-35 mT) magnetic excitations, respectively. Source data are provided as a Source Data file. **e**, **f**, and **g** show different states of the design after a second-stage optimization. **h** Energy analysis for the kirigami in Fig. S8f. Regions shaded in red correspond to analysis under constant positive (+35 mT) and constant negative (-35 mT) magnetic excitations, respectively. Source data are provided as a Source Data file.

In the second stage, we continue to fine-tune the first-stage design to make both compact states become energy minimum in the original problem. We fix the cutting pattern $\{\tilde{x}_i, \tilde{y}_i\}$ and optimize the magnetization orientation of each panel to minimize the original objective function

$$\min_{d_i} \bar{\mathcal{F}} = \ln[\mathscr{g}(S_0|S_-,\mathbf{B})^2 + \mathscr{g}(S_0|S_+,-\mathbf{B})^2 + \delta]\,. \tag{S48}$$

Since the cutting pattern is fixed, the configuration in the two compact states can be obtained before the optimization and remain the same during the whole optimization process. Therefore, by substituting the fixed configurations of different states into the objective function in equation (S48), it becomes a function with the magnetization orientation $\{d_i\}$ as the only design variable. We can then easily obtain the analytical gradient and efficiently solve this second-stage optimization with the SQP solver. As demonstrated in Fig. 6 and Fig. S8e-h, it enables us to obtain a two-way contractible design that can achieve physical equilibrium in both compact states.

Supplementary Note 7. Printing of the magnetic kirigami

The magnetic kirigami patterns are created using the Direct Ink Writing (DIW) method, employing ink prepared according to the procedure outlined in the Methods section. The ink is magnetized using a homemade magnetizer under a 1.5 T impulse magnetic field. The syringe is attached to a customized gantry 3D printer (Aerotech) featuring a 410 μm printing nozzle for the fabrication process. With an attached permanent magnet on the nozzle, the polarities of magnetized particles in the ink are aligned to the printed direction during extrusion (Fig. S9a). The kirigami panel magnetization is thus programmed by controlling the filament printing direction. Note that there is a magnetic field shield between the permanent magnet and the printing platform so that the magnetizations of printed filament remain unchanged when the printing nozzle is moving around. For a given optimized kirigami design (Fig. S9b), a printing pattern drawing is created with the encoded printing direction of each panel while considering the kirigami hinge dimensions. Then, CADFusion (Aerotech) is used to convert magnetic kirigami pattern drawings to G-codes for printing. The printing speed is set to 5 mm·s^{-1} with extrusion pressure being 200 kPa. The printed patterns are cured at 80°C for 36 h.

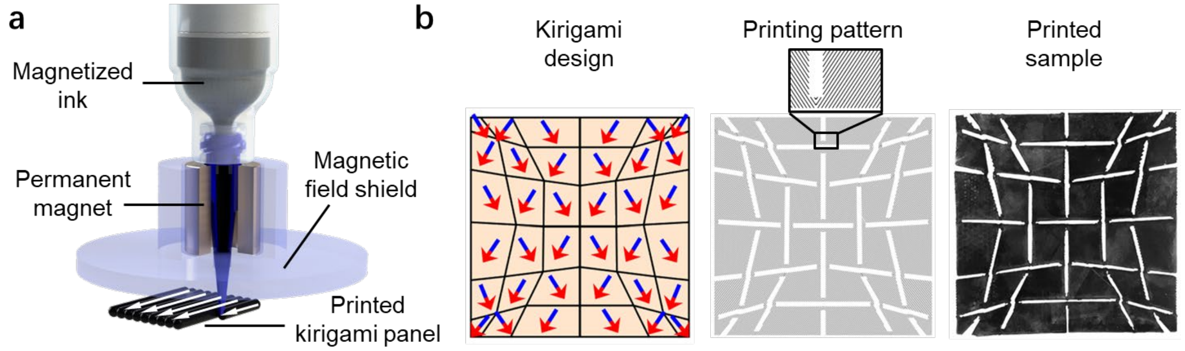

Supplementary Figure 9. Direct ink writing printing process of magnetic kirigami. **a** Schematic direct ink writing printing of magnetic composite for a panel with programmed magnetization. **b** The optimized kirigami design, the converted printing pattern, and the printed sample with the circular deployed state in Fig. 4a~d.

Supplementary References

1 Choi, G. P., Dudte, L. H. & Mahadevan, L. Programming shape using kirigami tessellations. *Nature materials* **18**, 999-1004 (2019).

2 Choi, G. P., Dudte, L. H. & Mahadevan, L. Compact reconfigurable kirigami. *Physical Review Research* **3**, 043030 (2021).

3 Zhao, R., Kim, Y., Chester, S. A., Sharma, P. & Zhao, X. Mechanics of hard-magnetic soft materials. *Journal of the Mechanics and Physics of Solids* **124**, 244-263 (2019).

4 He, L., Lou, J., Dong, Y., Kitipornchai, S. & Yang, J. Variational modeling of plane-strain hyperelastic thin beams with thickness-stretching effect. *Acta Mechanica* **229**, 4845-4861 (2018).

5 Nocedal, J. & Wright, S. J. *Numerical optimization*. (Springer, 1999).
