# Supplementary material for: Physics-aware differentiable design of magnetically actuated kirigami for shape morphing
Source: Nat Commun. 2023 Dec 21;14:8516. doi: 10.1038/s41467-023-44303-x (PMC10739944; doi:10.1038/s41467-023-44303-x)
Supplement: Supplementary file 11 — Description of Additional Supplementary Files [file 41467_2023_44303_MOESM11_ESM.pdf]

Description of Additional Supplementary Files

Supplementary Movie 1. Simulated shape morphing process with circular deployed shapes under different excitations corresponding to Fig. 4.

Supplementary Movie 2. Experimental results for shape morphing process with circular deployed shapes corresponding to Fig. 4c-d.

Supplementary Movie 3. Simulated shape morphing process with various deployed shapes in Fig. 5.

Supplementary Movie 4. Experimental results for the shape morphing process with a gobletlike deployed shape corresponding to Fig. 5g.

Supplementary Movie 5. Simulated two-way shape morphing process with a circular shape in the zero state, corresponding to Fig. 6a, 6c, and Fig.7a.

Supplementary Movie 6. Experimental results for the two-way shape morphing process with a circular shape in the zero state, corresponding to Fig. 6b and Fig.7a.

Supplementary Movie 7. Simulated two-way shape morphing process with a goblet-like shape in the zero state, corresponding to Fig. 6d, 6f, and Fig.7b.

Supplementary Movie 8. Experimental results for the two-way shape morphing process with a goblet-like shape in the zero state, corresponding to Fig. 6e and Fig.7b.
